# Comparison of chloroplast genomes and phylogenetic analysis of four species in *Quercus* section *Cyclobalanopsis*

**DOI:** 10.1038/s41598-023-45421-8

**Published:** 2023-10-31

**Authors:** Xiaoli Chen, Buyu Li, Xuemei Zhang

**Affiliations:** College of Life Sciences, China West Normal University, Nanchong, 637009 China

**Keywords:** Plant genetics, DNA sequencing, Sequence annotation

## Abstract

The identification in *Quercus* L. species was considered to be difficult all the time. The fundamental phylogenies of *Quercus* have already been discussed by morphological and molecular means. However, the morphological characteristics of some *Quercus* groups may not be consistent with the molecular results (such as the group *Helferiana*), which may lead to blurring of species relationships and prevent further evolutionary researches. To understand the interspecific relationships and phylogenetic positions, we sequenced and assembled the CPGs (160,715 bp-160842 bp) of four *Quercus* section *Cyclobalanopsis* species by Illumina pair-end sequencing. The genomic structure, GC content, and IR/SC boundaries exhibited significant conservatism. Six highly variable hotspots were detected in comparison analysis, among which *rpoC1*, *clpP* and *ycf1* could be used as molecular markers*.* Besides, two genes (*petA, ycf2*) were detected to be under positive selection pressure*.* The phylogenetic analysis showed: *Trigonobalanus* genus and *Fagus* genus located at the base of the phylogeny tree; The *Quercus* genus species were distincted to two clades, including five sections. All Compound Trichome Base species clustered into a single branch, which was in accordance with the results of the morphological studies. But neither of group *Gilva* nor group *Helferiana* had formed a monophyly. Six Compound Trichome Base species gathered together in pairs to form three branch respectively (*Quercus kerrii* and *Quercus chungii*;* Quercus austrocochinchinensis* with *Quercus gilva; Quercus helferiana* and *Quercus rex*). Due to a low support rate (0.338) in the phylogeny tree, the interspecies relationship between the two branches differentiated by this node remained unclear. We believe that *Q. helferiana* and *Q. kerrii* can exist as independent species due to their distance in the phylogeny tree. Our study provided genetic information in *Quercus* genus, which could be applied to further studies in taxonomy and phylogenetics.

## Introduction

*Quercus* L. is the most diverse genus in Fagaceae, with 430 species worldwide, which is one of the most widely distributed woody genera in Northern Hemisphere. Based on the morphology, molecular, and evolutionary history researches, *Quercus* genus was separate into two subgenera, namely *Quercus* and *Cerris*, including eight sections^1,2^. China is the second center of oak diversity and has identified and utilized the Fagaceae plants for the first time. *Quercus* section *Cyclobalanopsis* (ca.150 species) mainly distributed in tropical and subtropical regions in Asia, which was divided into six groups by morphological features^3^, and *Quercus austrocochinchinensis, Quercus kerrii,* and *Quercus rex* were considered to belong to the group *Helferiana* inside. The three species were clustered to a branch based on leaf epidermal features, but when using RAD-Seq data, they were dispersive and did not represented as monophyletic^4,5^, suggesting that the phylogeny location of these three species remained doubts. In addition, there were a series of transitional traits in the morphology of *Quercus helferiana* and *Q. kerrii*, showing high similarity with in morphology^3^. Wu et al. believed that these two species should be classified as the same species^6^, but subsequent research by Deng, M. found that the similarity of these two species is inconsistent in different populations^3^. The kinship between these two species therefore remains to be studied.

While it is a consensus for a long time that characters of the abaxial leaf surface and pollen provide valuable information for the species delimitation at infrageneric level^5,7,8^. However, when molecular sequence data were used to recognise (sub)sections/series, the result do not always conform to groups identified by means of traditional morphological classification within oaks^9^. For example, the research based on ITS sequences indicate that the species of compound trichome base (CTB) group of *Quercus* section *Cyclobalanopsis* converge into the same branchand with *Quercus* section *Cerris*, which is greatly different from the traditional classification of morphology^3^. Due to the similarities of leaf characteristics and gene introgression among different groups, despite lots of studies on morphological characteristics of *Quercus* section *Cyclobalanopsis*, more molecular evidence is needed for interspecific relationship and infrageneric phylogenetic status within *Quercus* genus.

Plastid exhibits key functions in plant growth and photosynthesis, and had independent genetic material, manifesting a tetrad structure^10–12^. Due to the maternal inheritance of the chloroplast genome (CPG), which was smaller in size, lower in nucleotide substitution rate, and more stable in structure compared to the nuclear genome, it exhibits conservative genetic variation^13–16^. CPGs can significantly enhance resolution at lower taxonomic levels and facilitate recovery of monophyletic lineages^17^, and are therefore considered ideal material in phylogenetics and population genetics^14,18–20^. In recent years, DNA sequencing technology has shifted to high-throughput, and CPGs of a large number of plants have been sequenced and published^21^, which was in turn used to identification and classification of plant^22–24^, lineage geography^25^ and phylogenetic relationship researches^26–28^. Due to the existence of overlap and mosaicism in important taxonomic morphological traits among the species of *Quercus* section *Cyclobalanopsis*^5^, molecular means such as chloroplast genomes can be used to explore intragroup interspecific relationships, identify species, and inform the implementation of species conservation strategies.

Currently, 50 CPGs for *Quercus* spp. could be queried in the National Center for Biotechnology Information (NCBI) database, 14 of which are from the sect. *Cyclobalanopsis*^29^. Here, we newly present CPGs sequences of four *Quercus* section *Cyclobalanopsis* species, including: *Quercus austrocochinchinensis, Quercus kerrii, Quercus helferiana,* and *Quercus rex.* Using these CPGs, we performed: (1) Structure and gene annotation; (2) Comparative genomics analysis; (3) Selection pressure and phylogenetic analysis. This study aims to investigate: Characteristics and differences of CPGs among the four species; Hypervariable regions for the CPGs studied; Phylogenetic status of *Quercus* genus species. Our study will enrich the molecular data for the phylogenetic study and conservation of endangered species in the *Quercus* section *Cyclobalanopsis*.

## Materials and methods

### Plant samples, DNA extraction and sequencing

Tender, unwounded leaf of 4 *Quercus* section *Cyclobalanopsis* species (*Quercus austrocochinchinensis, Quercus kerrii, Quercus helferiana,* and *Quercus rex*) were harvested from three provinces in China: Yunnan, Hainan and Guizhou. Silica gel was used to dry the materials collected. Voucher specimens were saved in China West Normal University (CWNU) and sample information was listed in Table1. The improved CTAB protocol was used to extract and purify total genomic DNA from leaf tissues (6 g per species)^30^. We used the high-quality genomic DNA to constructed a 400 bp Illumina Nova Seq library according to the manufacturer's protocol. Then the sequencing was performed on the Illumina Nova Seq PE150 platform, using pair-end strategies. Quality control on the raw data used FastQC^31^. Use Adapter Removal^32^ to leach the joint contamination at the 3'end; quality filtration by sliding window method. Sequencing information was provided in Table 1.

**Table 1:** Basic information of 4 *Quercus* section *Cyclobalanopsis* species.

Taxa	Voucher	Clean bases (G)	Average coverage (×)	NCBI accession number
*Q. austrocochinchinensis*	CHINA. Hainan, 19°7′21.648″ N, 109°9′36.828″ E, 613 m	2.11	92.7	OQ998918
*Quercus. helferiana*	CHINA. Guizhou, 25°3′47.559″ N, 106°23′1.311″ E, 932 m	2.17	83.3	OQ998919
*Quercus. kerrii*	CHINA. Hainan, 19°7′24.708″ N, 109°9′39.672″ E, 606 m	2.24	72.0	OQ998920
*Quercus. rex*	CHINA. Yunnan, 22°36′46.742″ N, 101°6′13.042″ E, 1595 m	2.29	59.9	OQ998921

### Chloroplast genome assembly, annotation and visualization

CPGs were assembled by following steps: Firstly, clean reads were assembled by GetOrganelle^33^, with the iterative k-mer sizes setting to 21, 45, 65, 85, and 105. Secondly, the assembled results were edited into circular sequences using Bandage^34^. Thirdly, the Geneious^35^ were using to adjust the initiations and find inverted repeat region. Assembled CPGs were annotated by Online website CPGAVAS2^36^, and the complete plastome sequence of *Quercus ningangensis* (NC_061582) as a reference. The intron/exon boundaries of annotation sequence were checked by Geneious. The CPG sequences and annotations were put in NCBI database. CPGs map were drawn on OGDRAW^37^.

### Genome structure and codon usage analyses

In order to understand the framework of whole chloroplast genomes, Geneious was used to identify the size, genes and GC content in CPGs. Then confirmed and visualized the boundaries between IRs/SCs by IRscope^38^. The totality of codons and RSCU (relative synonymous codon usage) values were calculated by CodonW with default parameters^39^.

### Sequence divergence and comparative analyses

The types of long sequence repeats (LSRs) were predicted by REPuter^40^, including type forward (F), type reverse (R), type complementary (C) and type palindromic (P), with parameters setting to: 30 bp for minimum repeat sequence, 3 for Hamming distance. In addition, MISA^41^ with parameters setting of ≥ 10 for type mononucleotides, ≥ 5 for type dinucleotides, ≥ 4 for type trinucleotides, and ≥ 3 for type tetranucleotides, pentanucleotides and hexa-nucleotides were applied to predicted SSRs quantity and types. Multiple sequence alignment of CPGs were performed in mVISTA^42^, selecting Shufe-LAGAN mode when analyzing with *Quercus gilva* (MG678009) as a reference. After alignment the sequence by MAFFT^43^ with default parameters, nucleotide diversity (Pi) values of CPGs evaluating were performed using DnaSP^44^.

### Selection pressure and phylogenetic analyses

KaKs_Calculator^45^ was adopted to calculated the rate of nonsynonymous mutation (Ka), synonymous mutation (Ks) in protein-coding genes. So that the results of Ka/Ks could be used to assesse the role of selection for each gene in CPGs of 11 *Quercus* species, seven species of which were downloaded from NCBI (Supplementary Table S7).

For the purpose of acquainting with the phylogenetic relationships, phylogenetic tree of *Quercus* genus were implemented using Bayesian (BI) analysis methods, based on the CPG data. The CPG sequences required for the phylogenetic analysis are shown in the Table S7, including 27 Fagaceae species downloaded from NCBI. Apply all selected CPG sequences to MAFFT^43^ to align. Later MrBayes^46^ was utilized to carry out the BI tree analysis on the basis of following processes: infer the best-fit nucleotide substitution model (GTR + F + I + G4) by Modeltest^47^ and PAUP^48^; Run 6,000,000 generations in Markov chain Monte Carlo (MCMC) analysis; Sample the trees each 1,000 generations, and ignore the initial 0.25 as burnin fraction.

## Results

### Characteristics of the CPGs

**Figure 1:**
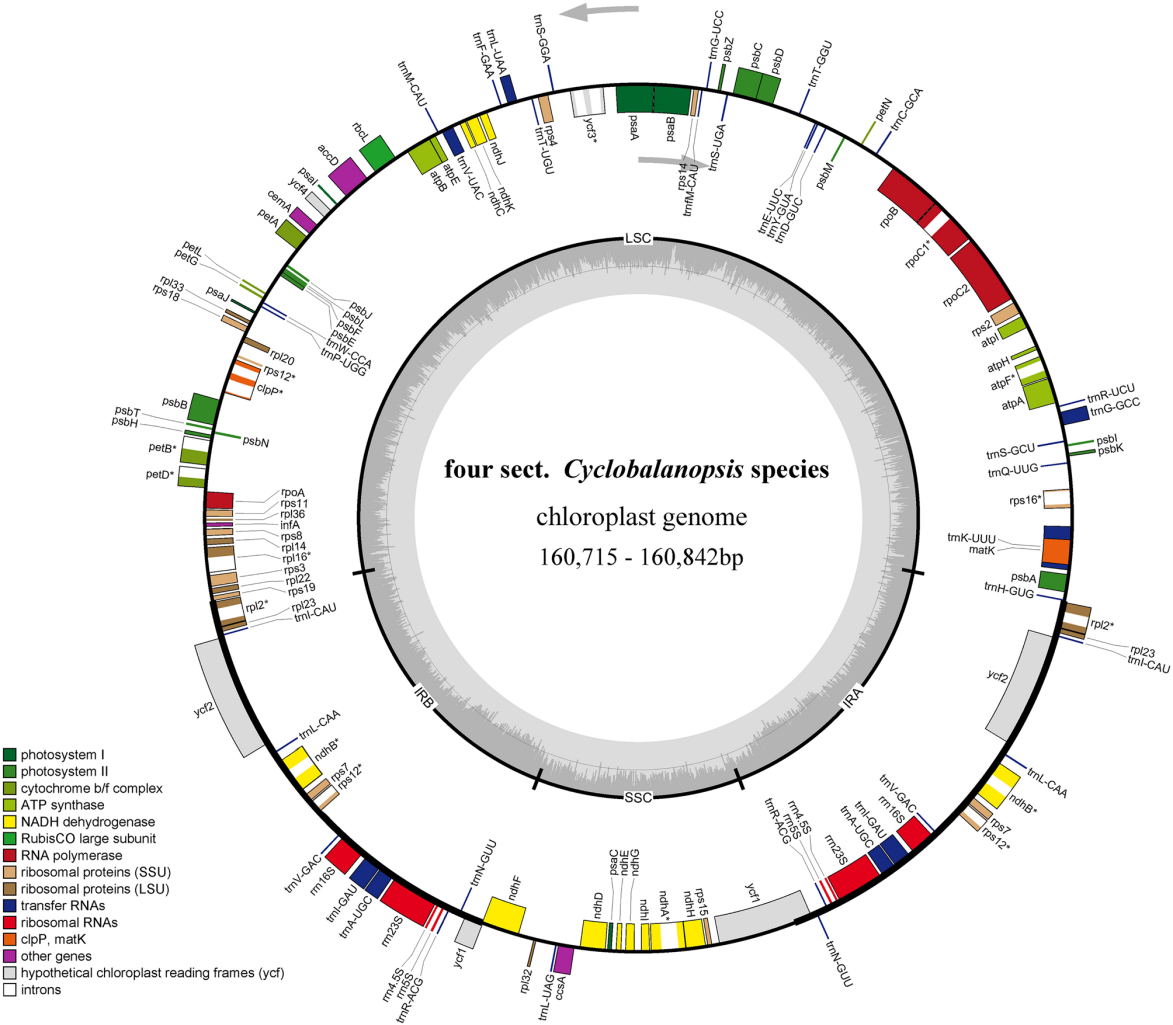
CPGs Gene map of four *Quercus* section *Cyclobalanopsis* species. Diferent colors refer to different functions of genes. The chloroplast genome map was draw using OGDRAW v1.3.1 (https://chlorobox.mpimp-golm.mpg.de/OGDraw.html).

The length of 4 CPGs assembled scoped from 160,715 bp in *Q. kerrii* to 160,842 bp in *Q. rex*. All the structures manifest same circular quadripartite tetrad, comprising of 2 single-copy areas (LSC, SSC) and a couple of inverted repeats (IRs). The length of each region was shown in Table 2. The GC content of general sequences was 36.9% for all samples. Besides, the GC content in IRs lead to 42.8%, which was greater than that in LSC and SSC areas (34.8% and 31.1%). Additionally, all the four CPGs encoded 131 genes, namely 86 CDS, 37 tRNA and eight rRNA, and it should be noted that 18 (seven CDS, seven tRNA and four rRNA) of these were iterant in the IRs. Among all of the genes, 15 have an intron and three genes (*rps12*, *clpP*, *ycf3*) with two. The specific distribution and function of the genes were shown in Fig. 1, Supplementary Table S1.

**Table 2:** A summary of the statistics for the CPGs of 4 *Quercus* sect. *Cyclobalanopsis* species.

Species	*Quercus kerrii*	*Quercus austrocochinchinensis*	*Quercus helferiana*	*Quercus rex*
Genome size (bp)	160,715	160,768	160,801	160,842
Length of LSC (bp)	90,135	90,231	90,216	90,281
Lengh of IRs (bp)	25,841	25,835	25,840	25,839
Length of SSC (bp)	18,898	18,867	18,905	18,883
Number of genes	131	131	131	131
protein-coding genes	86	86	86	86
tRNA genes	37	37	37	37
rRNA genes	8	8	8	8
GC content (%)	36.9	36.9	36.9	36.9
GC content of LSC (%)	34.8	34.8	34.8	34.8
GC content of IRs (%)	42.8	42.8	42.8	42.8
GC content of SSC (%)	31.1	31.1	31.1	31.1

**Figure 2:**
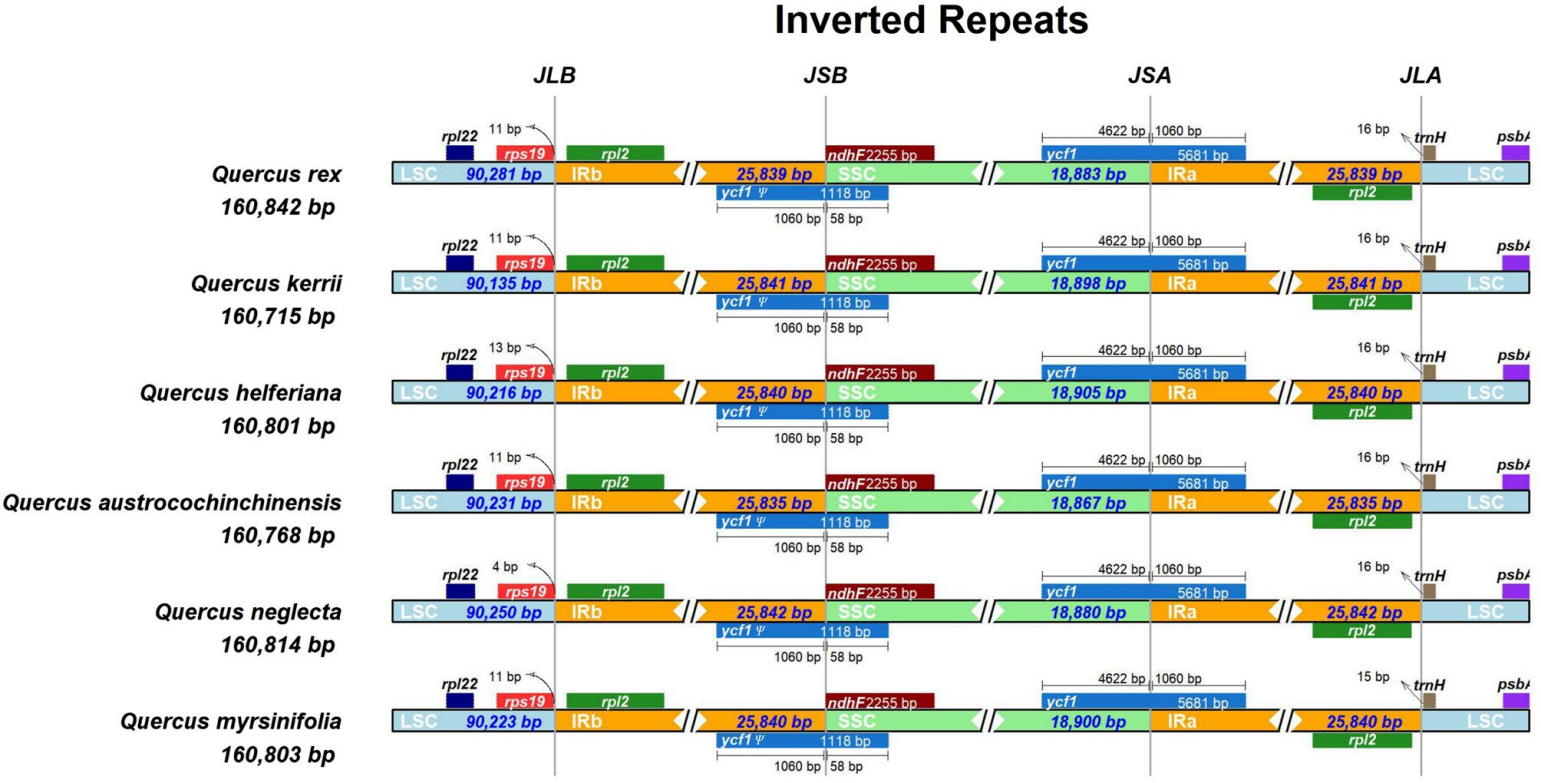
CPGs boundary comparison in six *Quercus* section *Cyclobalanopsis* species. Genes shown above the lines were transfered in reverse and those displayed below were transfered forward.

Figure 2 gave the results of CPGs boundary comparison in six *Quercus* section *Cyclobalanopsis* species, which could show the borderlines of the IRs and SCs regions in CPGs. The junction of LSC and IRb (*JLB*) laid in IGS (intergenic spacer) of *rps19* and *rpl2* gene. Most samples had 11 bp shift away from the borderline for *rps19* gene in JLB, except *Q. helferiana* and *Q. neglecta*, which had 13 bp and four bp shift respectively. Moreover, the demarcation of LSC and IRa was situated in the IGS of *rpl2* and *trnH* gene, with the *trnH* gene shifting 15 or 16 bp from JLA. IRa/SSC boundary (*JSA*) was reposed within gene *ycf1.* What should be noted was that the 5’ end of gene *ycf1* standed in the IRa area but the 3’ end standed in SSC area, therefore created a 5’ end pseudogene (*ycf1Ψ*) in the IRb in all CPGs compared, resulting in all IRb/SSC (*JSB*) boundaries lying within the pseudogene ycf1Ψ.

The codon usage analysis summarized in Table 3. According to the results, sequence sizes range of extracted protein-coding genes were 64,359–64,377 bp in four *Quercus* section *Cyclobalanopsis* species; 21,453–21,459 codons were encoded. The ENC (Effective Number of Codons) value was between 49.93 and 49.97. The FOP (Frequency of Optimal Codons) value was 0.353 in *Q. kerrii* and 0.354 in other three samples. The GC content was between 37.93 and 37.95%. The codon preference indexes of the four species varied slightly, indicating that they had similar codon usage. The GC3 of four species ranged between 29.85 and 29.88%, indicating that they prefer codons ending with A/U.

**Table 3:** Codon preference index of four species of *Quercus* section *Cyclobalanopsis.*

Index	*Quercus. kerrii*	*Quercus. austrocochinchinensis*	*Quercus. helferiana*	*Quercus. rex*
Length (bp)	64,359	64,359	64,359	64,377
Codon number	21,453	21,453	21,453	21,459
Effective number of codons	49.94	49.97	49.96	49.93
Codon adaptation index	0.166	0.166	0.166	0.166
Codon bias index	− 0.100	− 0.100	− 0.100	− 0.100
Frequency of optimal codons	0.353	0.354	0.354	0.354
GC content (%)	37.93	37.95	37.94	37.93
GC1 content (%)	46.05	46.05	46.06	46.03
GC2 content (%)	37.82	37.84	37.82	37.83
GC3 content (%)	29.92	29.95	29.93	29.92

**Figure 3:**
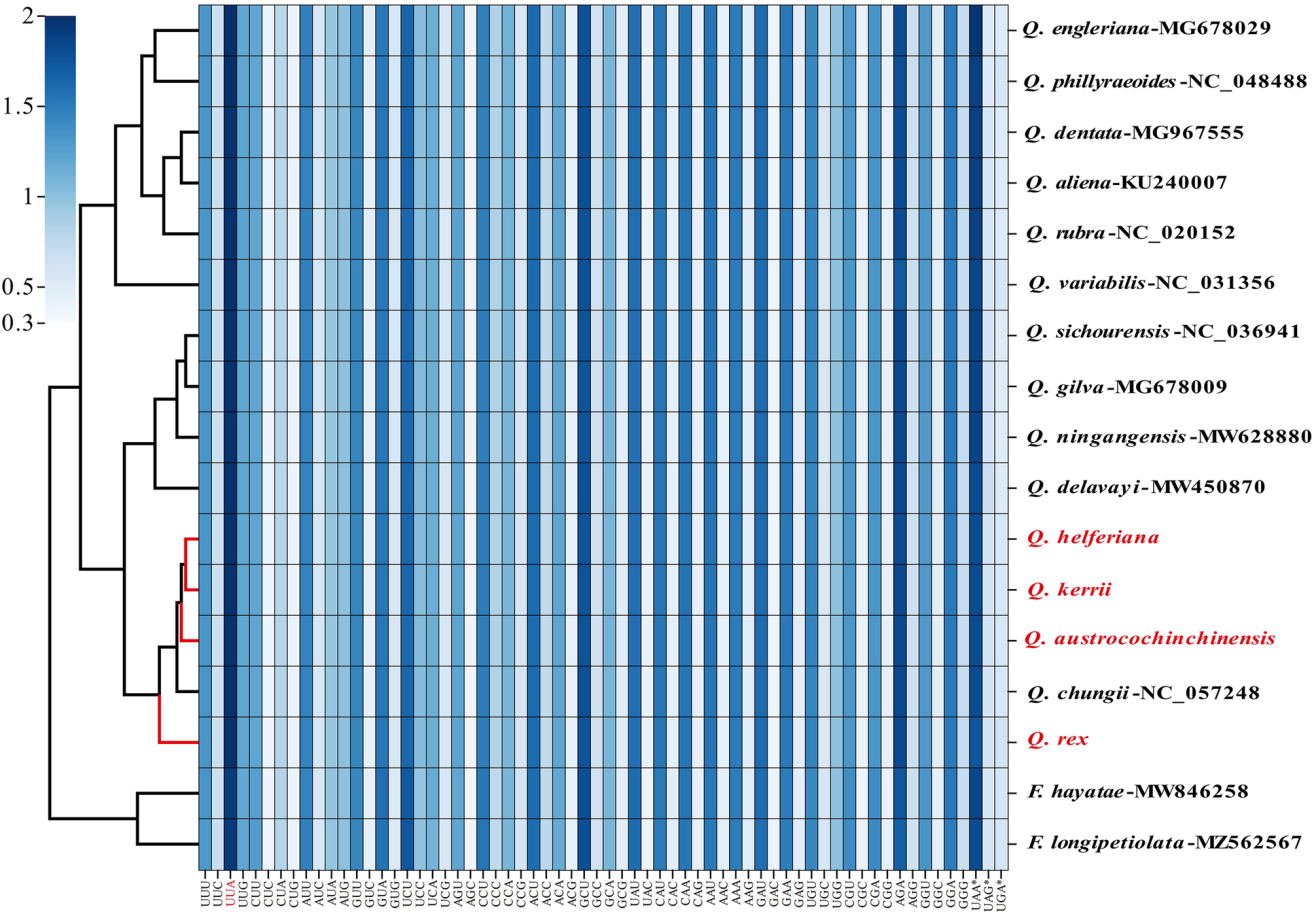
RSCU ratios of CDS genes for CPGs in four species and its sibling species of Fagaceae*.* (*) indicated the stop codon. The red font means the four species we studied.

The CDSs of 17 CPGs (four newly sequenced and 13 species of Fagaceae released in NCBI) were extracted using Geneious. Subsequently, based on the extracted sequences, the ratio of RSCU in all samples were calculated and clustered. The results showed in Fig. 3, Supplementary Table S2. We found that:

Leucine (Leu) encoded with the maximum number of codons, arranging from 2044 to 2268, with the number of isoleucine (Ile 1699–1892) following. The minimum number of codons (213 to241) presented in Cysteine (Cys). (2) The (RSCU) values varied marginally among the CDSs of 17 species. 31 codons were frequently manipulated since RSCU > 1, and the remaining codons were less frequently used as their RSCU ratios were less than 1. (3) The frequently used codons include: UUA, AGA, UAA(*), GCU, UCU, GAU, ACU, and the codon usage frequency of UAC, CUC, CGC, CUG, AGC, and GAC is on the low side. Thereinto the UUA codon showed a bias in 17 CPGs due to its highest usage. No usage frequency bias (RSCU = 1) showed in the starting codons of AUG and UGG, which encoded methionine (Met) and tryptophan (Try).

### Repeated sequences analysis

**Figure 4:**
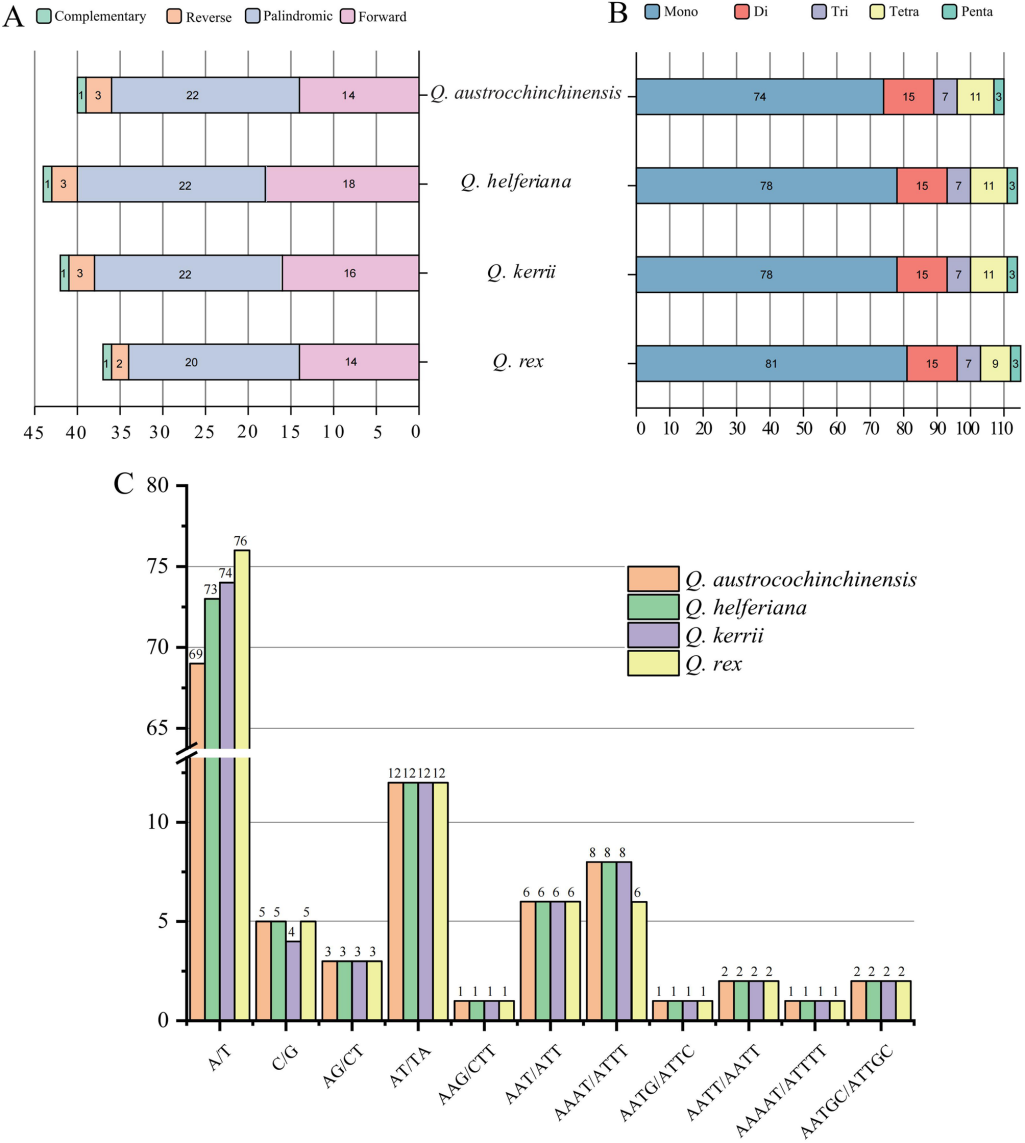
Distribution of repeats in four samples of Quercus section Cyclobalanopsis. Part A-type and number of LSRs. Part B-distribution of SSRs types. Part C-the number of SSR units detected.

A total of 163 LSRs were identified among the four CPGs examined. As a whole, the amount of LSRs identified in every CPG was scoping from 37 in *Q. rex* to 44 LSRs in *Q. helferiana*. Thereinto, 14–18 were type F, 20–22 were palindromic repeats, and the number of type R was two in *Q. rex* when other three species were three (Fig. 4A. Supplementary Table S3). Just one complement repeat was filtrated from four species. Among these repeats, the longest repeat was 56 bp in every species, and the most common length of repeats was 30 bp. 44.5% LSRs located in IGS, and 23.5% were found in the intron region. About half repeat sequences (46.8%) were distinguished in the IR areas (Table S3).

The total quantitiy of SSRs identifed in the CPGs of four *Quercus* section *Cyclobalanopsis* species was 453, ranging from 110 in *Q. austrocochinchinensis* to 115 in *Q. rex*, among which 74–81 were type mono-, 15 were type di-, seven were type tri-, 9–11 were type tetra-, and three were penta- (Fig. 4B). The most universal unit of SSRs was A/T (mono-), whose amount ranged from 69 to 76, far higher than in the other types. 68% of SSRs were type mononucleotide made up of unit A/T and C/G. What's more, most of the SSRs (70.8%) were located in the IGS (Supplementary Table S4). All the type din- comprised multiple copies of unit AT/TA and AG/CT (Fig. 4C). The type of hexanucleotide was not detected in all species. Taken as a whole, no significant distinction in the number of SSR units among the four species, except the slight diferences in unit of mono- and penta-.

### Sequence divergence, hotspots and selection pressure estimation

**Figure 5:**
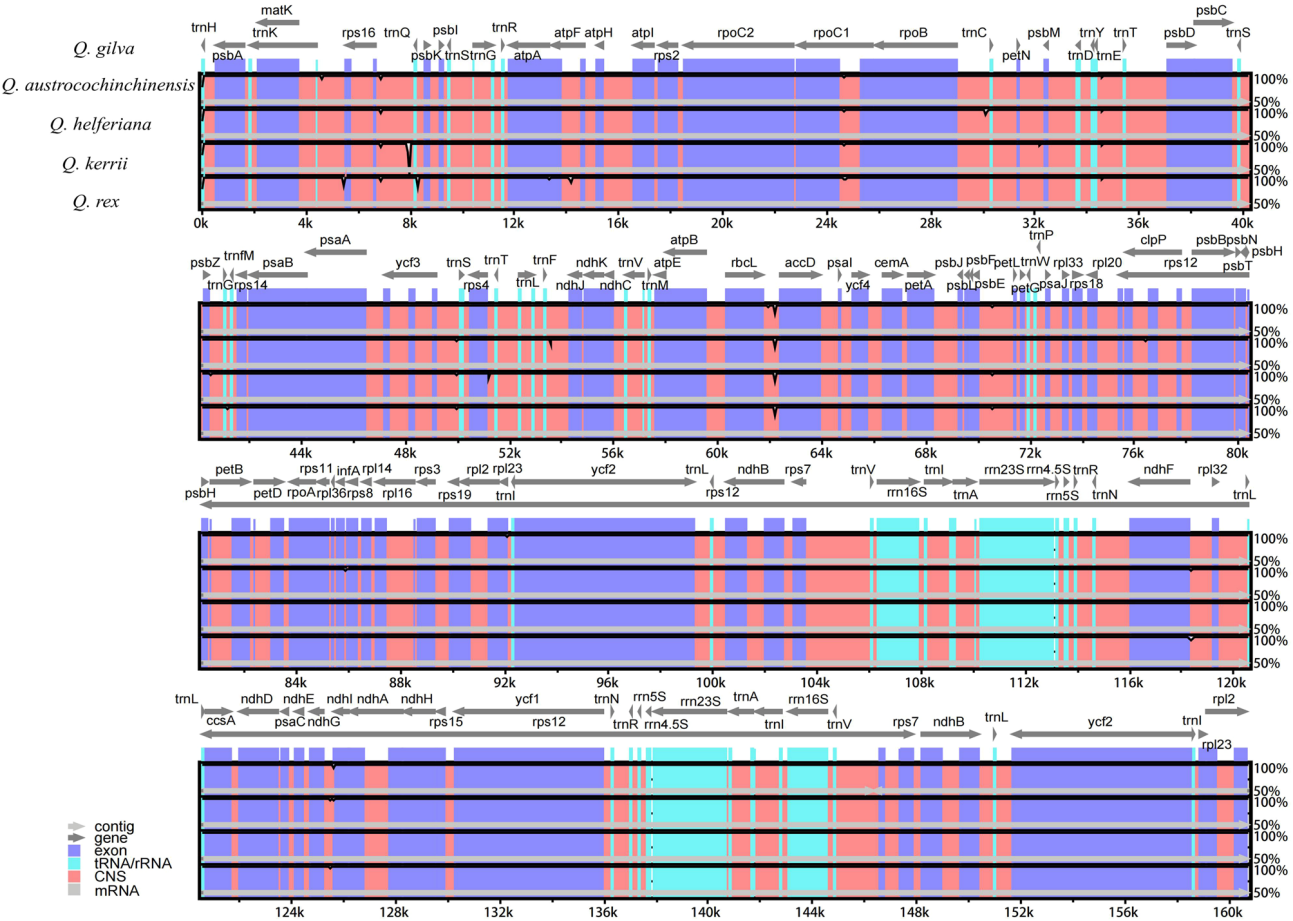
Sequence alignment of the CPGs of four *Quercus* section *Cyclobalanopsis* species. The *Quercus. Gilva* (MG678009) was used as reference.

CPGs Comparative analysis could be seen in Fig. 5, revealing that high sequence similarity among the four sect. *Cyclobalanopsis* species. Sequences in noncoding areas were more variant than in coding areas generally. Besides, the level of sequence divergence in SCs areas were evidently higher than that in IR areas. We found eight intergenic regions were in a high degree of variation, of which seven were located at LSC areas as follows: *psbA/trnH*, *rps16/trnK*, *trnQ/rps16, trnE/trnT*, *rbcL/accD*, *psbE/petL, ndhF/rpl32*. One located at SSC areas, namely *ndhI/ndhG*. Other than aforementioned areas, the intron area of *rpoC1* showed high level of sequence divergence too.

**Figure 6:**
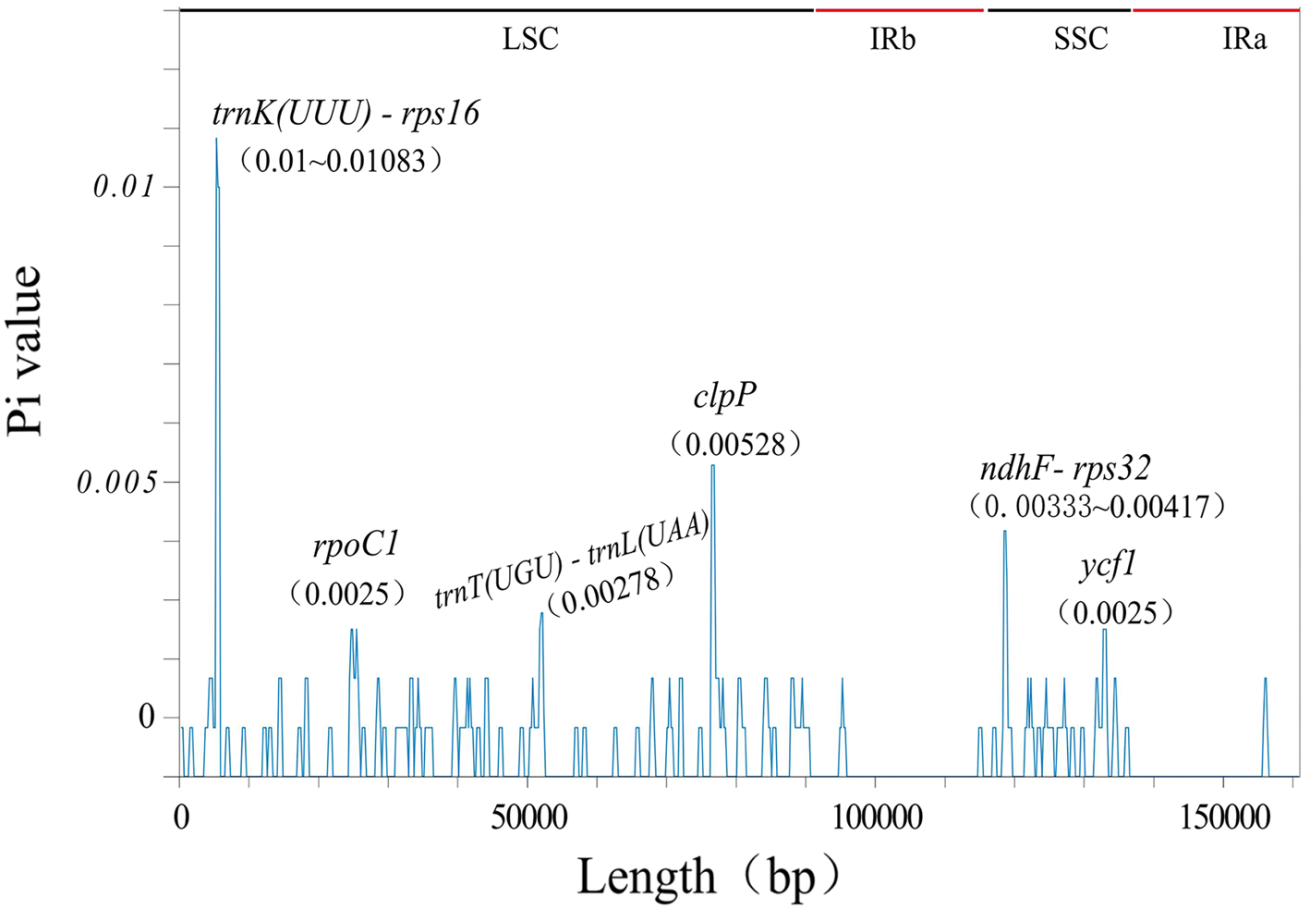
Pi values in the multiple alignments of 4 CPGs, details in Supplementary Table S5.

Window length setting to 600 bp, we calculated the nucleotide diversity values to elucidate levels of diversity for all CPGs assembled in this study. The Pi values were recorded in Supplementary Table S5, ranging from 0 to 0.01083, with 0.00041 on average.When the amount of polymorphic loci outweighed the sum of mean and twofold standard deviation, the region is defined as a highly variable region^49^. Ultimately, six hotspots (Pi > 0.0022) were discovered, coding and nocoding regions each accounting for half. The greatest Pi value (0.01083) appeared in the region between gene *trnK-UUU* and *rps16*. The distribution of highly variable regions was shown in Fig. 6. In general, these regions were not located at the IR areas but all at the SC areas, which reflected an identical pattern of CPG structural variation.

**Figure 7:**
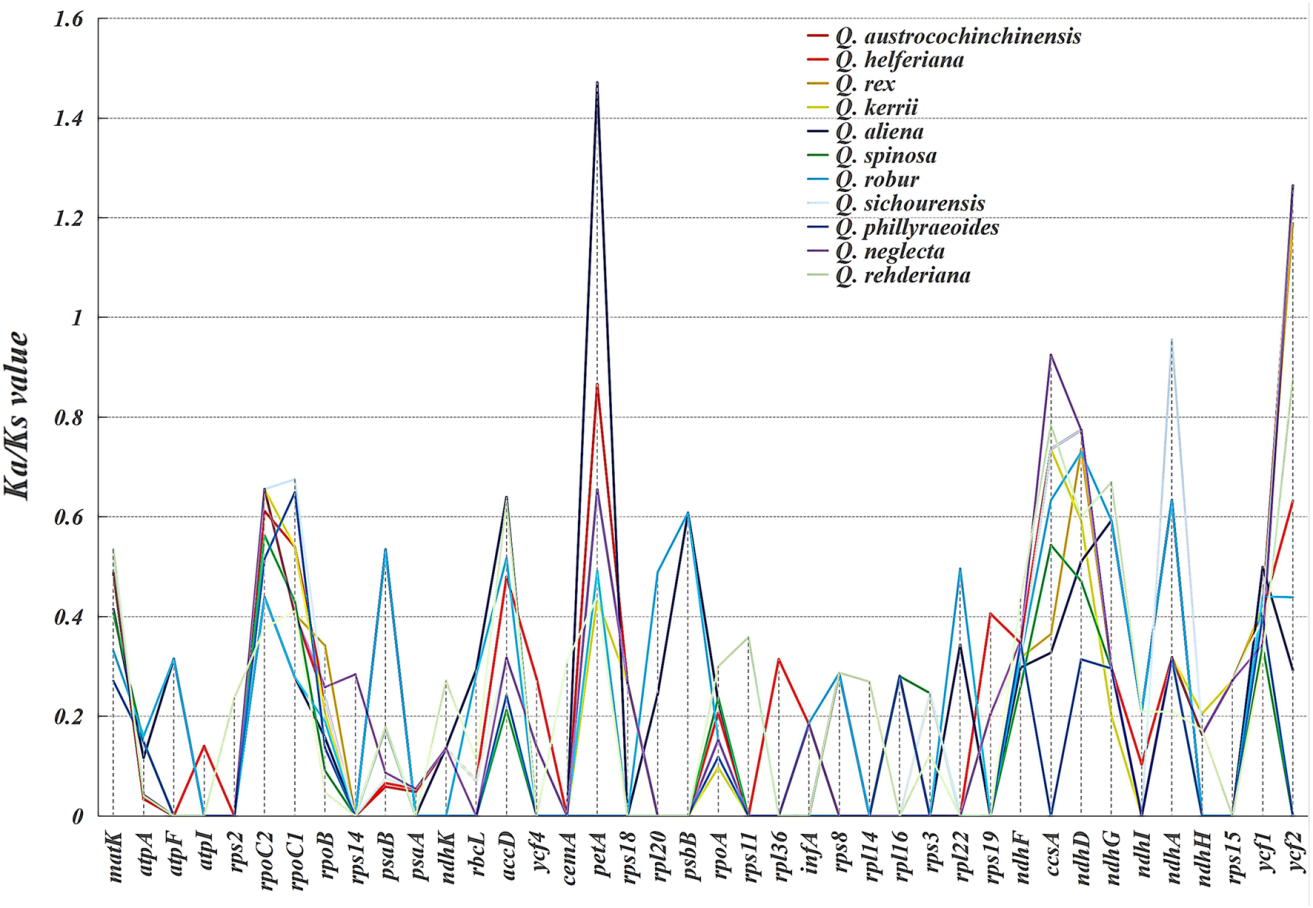
Ka/Ks of 40 protein-coding genes (details in Supplementary Table S6) in 11 *Quercus* CPGs.

To estimate the role of selection of the *Quercus* section *Cyclobalanopsis* species, Ka and Ks values of 79 unique CDS were calculated in 11 CPGs using *Quercus chenii* as a reference. The Ka/Ks values were simply calculated and recorded in the Supplementary Table S6, ranging from 0 to 1.471. Among which 40 protein-coding genes showed significance (Fig. 7) in 11 species. Based on the calculation results, we speculated that the purification selection may affect on most protein coding genes, as their Ka/Ks values were less than 1. At the same time, when Ka/Ks > 1 demonstrated that the positive selection was working on the genes. Therefore we identified two genes were under the positive selection, namely *petA* gene in *Q. aliena,* and *ycf2* gene in *Q. austrocochinchinensis, Q. rex, Q. kerrii, Q. sichourensis,* and *Q. neglecta.*

### Phylogenetic relationships

**Figure 8:**
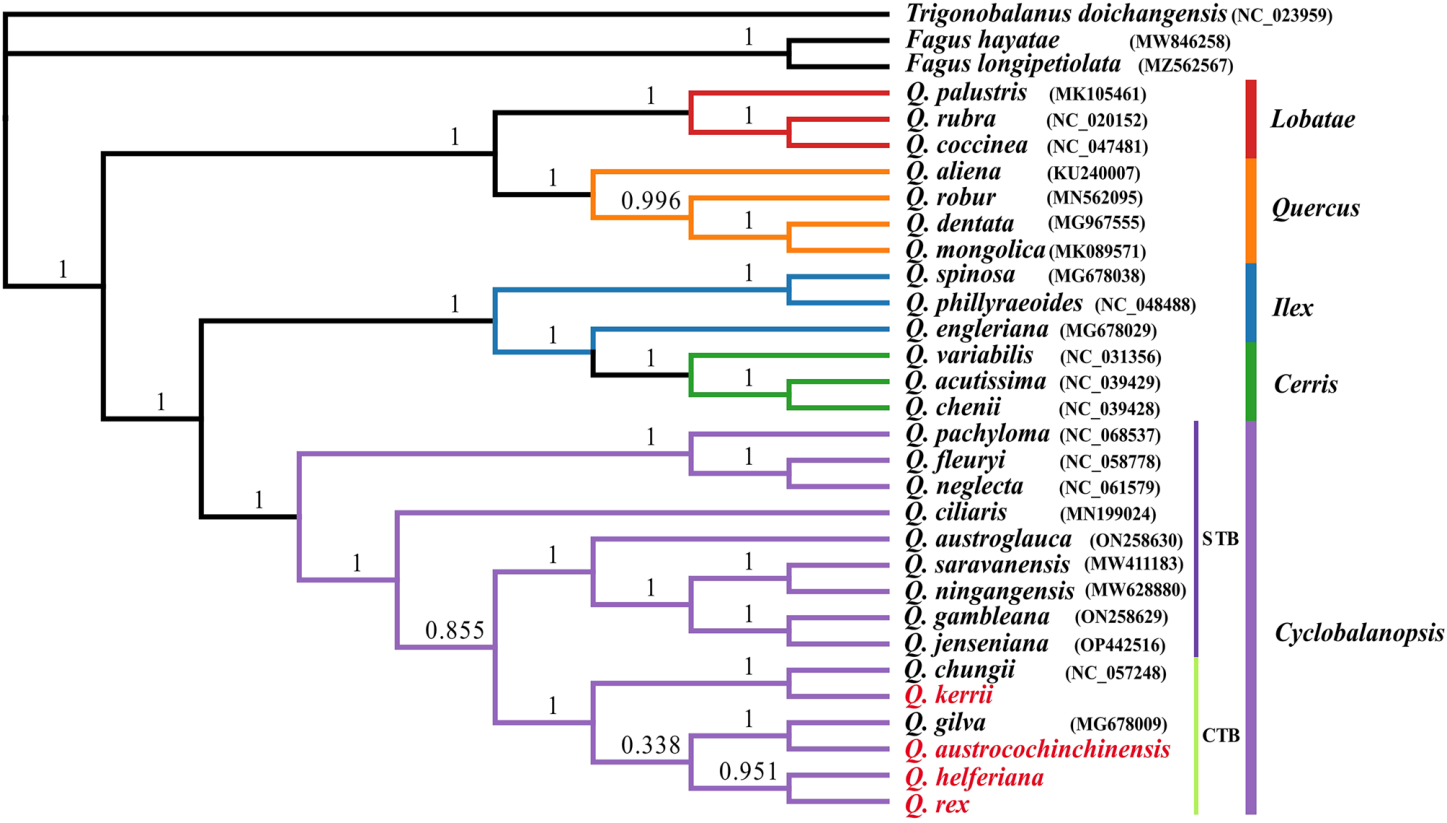
Bayesian (BI) analysis phylogenetic tree among 31 CPGs of Fagaceae species. Values above the branch represented bootstrap support.

Resorting to approaches BI, the phylogenetic relationships were reconstructed among the members of the four CPGs sequenced in this study and closely related species in *Quercus* genus, according to the whole chloroplast genome data. The *Trigonobalanus doichangensis* (NC_023959) was used as the outgroup. A total of 31 taxa were used, and the reconstructed phylogeny tree was shown in Fig. 8, and most branches obtained high support bootstrap values. Genus *Trigonobalanus* and *Fagus* located at the base of the phylogeny tree. Two distinct clades were recognized among all *Quercus* species analysised: the first clade consisted of two sections (four species in *Quercus* and three species in *Lobatae*). Another clade was divided into two nodes, including three sections, namely *Cyclobalanopsis*, *Cerris* and *Ilex*. In section *Cyclobalanopsis*, the species were divided to STB (Single-celled Trichome Base) and CTB (Compound Trichome Base). All the CTB species were clustered into a single branch including the four species we studied.

## Discussion

### CPG architectures in four *Quercus* section *Cyclobalanopsis* species

Four species CPGs were successfully assembled of *Quercus* Section *Cyclobalanopsis* in the present paper. The size of four CPGs (ca. 160 kb) conformed to the photosynthetic land plant plastid chromosomes, whose size varied from 120 to 160 kb^50^. The same quadripartite circular structure were found in the four assembled CPGs and other *Quercus* species^51–53^. Overall GC content had no distinction within the four species. After CPGs comparison, it was found that the totality, order, and function of genes were highly conservative in genus *Quercus*, which were also in accordance with most Fagaceae species^25,54,55^, evidencing a highly conservative CPG construction in *Quercus* Section *Cyclobalanopsis*.

Due to the duplicative nature of the IR reduced the substitution rate within this region, therefore it was of great significance to analyze the contraction and expansion of IRs in evolutionary researches^56^. In addition, the IR regions were vital in stabilizing the structure of the CPGs, which were also the main factor affecting the total length^57,58^. The results showed that boundaries of four areas in the CPGs were conserved in six *Quercus* section *Cyclobalanopsis* species. The IRs/SCs boundary of all species compared in this study were located within similar positions except for slight difference in *JLB,* whose displacements from *rps19* presented subtle variations in different species. Most of the compared species found no significant expansion or contraction in the IR regions except the *Quercus neglecta,* which had a only four bp displacement between the *JLB* and *rps19*. The conservatism of *Quercus* section *Cyclobalanopsis* was demonstrated by the relatively constant length of CPGs and the minor variations in their region borders, as the same conditions with other *Quercus* species^25,52^.

Codon usage bias was a natural phenomenon caused by mutation, natural selection, genome composition, etc^59–61^. In the PCGs of four cp genomes, total 64 codons were detected, encoding 20 amino acids. We could tell from the values of RSCU and content of GC3 that the bias in codon usage towards A/U at the third position, a phenomenon that is widespread in angiosperms^62–65^.

### Large repeats and simple sequence repeats

Dispersed in CPGs, long repeat sequences played an significant role the genomic inheritance, variation and the evolution of species^50,57,66^. Our study identified a total of 163 LSRs with palindromic being the most common type. The variations observed in CPGs could partially attributed to the differences in the number and types of LSRs^67^. Therefore, due to their genetic variations, LSRs can potentially provide valuable information for researches of phylogenetic relationship and population genetics. After analysis, it was found that the repeat sequences of this study were in accord with the general pattern above: about half (43.2–46.3%) of LSRs were identified in IGS, following by the coding regions and introns. Current studies had suggested that most repeats in CPG were situated in the IGS, comparing to the coding regions^15,68^. SSR had been extensively studied as a kind of effective molecular marker in various fields such as discrimination, breeding, conservation and phylogenetic research at the species and population levels^69–71^. A strong A/T bias, SC regions concentration (90.9–91.3%), and IGS concentration (69.6–72.7%) were detected in SSRs of four *Quercus* section *Cyclobalanopsis* species, similar to other *Quercus* genus species^29,72^. The numbers and types of SSRs varied slightly in *Quercus* genus but extensively in other families^73–75^. The numbers of SSRs were almost identical between *Quercus* section *Cyclobalanopsis* and section *Cerris*^74^, so we speculated that such case might imply that the two sections were phylogenetically more closely related.

### Conservatisms, highly variable regions and selection pressure estimation

We compared the whole sequences of CPG in four species with *Quercus gilva* as the reference. The results indicated that there were differences in the degree of variation between regions of CPGs, with the single-copy (SC) regions having higher variation than IR regions, simultaneously the IGS regions having higher variation than coding regions. Same phenomena were found in other *Quercus* species^51,52,76,77^. The copy-dependent repair mechanism of CPGs could guarantee the stability of IRs construction and thereby advance the steadiness and conservation of genomes, which possibly explain the different degree of variation between IRs and SCs. In addition, due to natural selection, the coding areas tend to exhibit higher conservation than the noncoding areas^78–80^. The gene regions of high variability we found (namely *rpoC1*, *clpP* and *ycf1*) in both sequence divergence analysis and nucleotide variability (pi) assessment could be used to develop DNA barcodes, conduct species identification and systematic classification^81^. Out of the highly variable regions identified, the *ycf1* gene^82^ and two IGS regions: *trnH-psbA*, *trnK-rps16* had already been selected as practicable barcode for plants^83–85^.

In our study, most of the Ka/Ks values were less than 1 or not available, suggesting that the emergence frequency of synonymous nucleotide substitution was more than that of non-synonymousnucleotide substitution due to the purify selection process^86,87^. We conjectured that positive selection was operating only in two genes: *petA* in *Q. aliena* and the *ycf1* in multiple *Quercus* taxa. The *ycf1* was indicated to contain multiple SSRs in many taxa and it was claimed that these SSRs were undoubted in detecting population-level polymorphisms and could also be used to compare phylogenetic relationships at the genus level or higher taxonomic levels^19,73^. Whether these divergence hotspots found in the above analysis could be utilized for DNA barcodes or estimating taxonomic evolution in genus *Quercus* needs more further researches.

### Inference of phylogenetic relationship

Due to the complex evolutionary issues such as convergent evolution, extensive hybridization, and serious hybridization introgression in the *Quercus* genus, great challenges remain in the phylogenetic relationship research of Oak trees^1,88,89^. CPGs have been demonstrated considerable utility in addressing the phylogeny relationships of angiosperms^90^. The phylogenetic trees we reconstructed based on CPGs indicated two major clades corresponding to geographic distribution: sections of *Quercus* and *Lobatae* constituted a “New World Clade” (subgenus *Quercus*), while the sections of *Cyclobalanopsis, Cerris* and *Ilex* forming an “Old World Clade” (subgenus *Cerris*)^9,89,91^. The section *Ilex* was paraphyletic, nested into the lineage formed by section *Cerris*, which was similar to the results based on plastid genome but differed from the phylogenetic relationships inferred from RAD-seq data^29,88,92^.

The morphological studies found that the four species we studied possessed compound trichome base (CTB) so that clustered into a single branch with other CTB species, distincting to the group STB (Single-celled Trichome Base)^5,93^, similar to the results of our phylogenetic study based on the CPGs. In the CTB group, *Q. kerrii* and *Q. chungii* clustered into a clade that diverging before the other four species, which had simple uniseriate thin-walled trichomes, distinct from other CTB species^5^. *Q. austrocochinchinensis* then clustered with *Q. gilva* into sister groups, which differed from the clustering results of RAD-seq data^4,94^. *Q. helferiana* and *Q. rex* gathered together, they all possessed Fasciculation trichomes^5^. Deng^3^ divided the CTB species into two groups based on their comprehensive morphological characteristics, namely group *Gilva* (containing *Q. chungii* and *Q Gilva*) and group *Helferiana*, including the four species we studied. From our results, we can see that neither of these two groups had formed a monophyly, and there were mosaics between these species. *Q. helferiana* and *Q. kerrii* were far apart in the phylogeny tree, so we believe that they can exist as independent species. Nevertheless the interspecific relationship within the four species remained some controversies: for instance, the *Q. kerrii* and *Q. austrocochinchinensis* gathered for a monophyletic sister branch in multiple studies, different from our BI tree. The *Q. rex* was thought to be the base of 4 species, but in our analysis the *Q. kerrii* differentiated firstly^4,5^. Due to the presence of one node with a low support rate (0.338) in the phylogeny tree, the interspecies relationship between the two branches differentiated by this node was still unclear. The continued advancements of sequencing techniques will allow for the inclusion of more taxa and samples in future studies, facilitating further exploration of the interspecific relationships and phylogenomics of the *Quercus* section *Cyclobalanopsis.*

## Conclusions

Chloroplast DNA has the characteristics of conservation and uniparental inheritance, which is of great significance for study in genetic diversity, population structure, and evolutionary relationships. In the present study, we completed the CPG basic analyses of four species in *Quercus* section *Cyclobalanopsis* and compared them with the genomes of other oak trees. Despite the overall conservation of CPG structure and gene content were obviously found, distinct sequence divergences were uncovered in alternating regions of these genomes among the studied species. The findings provide three genes including *rpoC1*, *clpP* and *ycf1* as DNA barcode for future studies of species identification and systematic classification. The phylogenetic analysis based on CPG data suggested: all *Quercus* species were divided into two categories, and consistent with the groups divided by morphology (STB and CTB); *Q. helferiana* and *Q. kerrii* were far apart in the phylogeny tree, so we believe that they can exist as independent species. In addition, the four species we studied, along with *Q. chungii* and *Q. gilva*, clustered into one branch with a bootstrap support rate of 1. Therefore, these six species exhibit close phylogenetic relationships in both morphology and molecular aspects, and should be classified into a group. In a word, the findings obtained will facilitate further investigations into the taxonomy, phylogenetic evolution and preservation of *Quercus* genus.

### Supplementary Information

Supplementary Table S1.

Supplementary Table S2.

Supplementary Table S3.

Supplementary Table S4.

Supplementary Table S5.

Supplementary Table S6.

Supplementary Table S7.

## Data Availability

The datasets generated and/or analysed during the current study are available in the [National Center for Biotechnology Information] repository, [Accession Number: OQ998918, OQ998919, OQ998920, OQ998921].

## Abbreviations

RAD-Seq: Restriction-site associated DNA sequencing
ITS: Internally Transcribed Spacer
CPG: Chloroplast Genome
CTAB: Cetyltrimethylammonium Bromide
LSC: Large single-copy
SSC: Small single-copy
IR: Inverted repeat
RSCU: Relative Synonymous Codon Usage
LSRs: Long Sequence Repeats
SSRs: Simple Sequence Repeats
BI: Bayesian inference
CDS: Coding sequence
IGS: Intergenic Spacer
